# From landraces to improved cultivars: Assessment of genetic diversity and population structure of Mediterranean wheat using SNP markers

**DOI:** 10.1371/journal.pone.0219867

**Published:** 2019-07-15

**Authors:** Rubén Rufo, Fanny Alvaro, Conxita Royo, Jose Miguel Soriano

**Affiliations:** Sustainable Field Crops Programme, Institute for Food and Agricultural Research and Technology (IRTA), Lleida, Spain; USDA-ARS Southern Regional Research Center, UNITED STATES

## Abstract

Assessment of genetic diversity and population structure in crops is essential for breeding and germplasm conservation. A collection of 354 bread wheat genotypes, including Mediterranean landraces and modern cultivars representative of the ones most widely grown in the Mediterranean Basin, were characterized with 11196 single nucleotide polymorphism (SNP) markers. Total genetic diversity (*H*_*T*_) and polymorphic information content (PIC) were 0.36 and 0.30 respectively for both landraces and modern cultivars. Linkage disequilibrium for the modern cultivars was higher than for the landraces (0.18 and 0.12, respectively). Analysis of the genetic structure showed a clear geographical pattern for the landraces, which were clustered into three subpopulations (SPs) representing the western, northern and eastern Mediterranean, whereas the modern cultivars were structured according to the breeding programmes that developed them: CIMMYT/ICARDA, France/Italy, and Balkan/eastern European countries. The modern cultivars showed higher genetic differentiation (*G*_*ST*_) and lower gene flow (0.1673 and 2.49, respectively) than the landraces (0.1198 and 3.67, respectively), indicating a better distinction between subpopulations. The maximum gene flow was observed between landraces from the northern Mediterranean SPs and the modern cultivars released mainly by French and Italian breeding programmes.

## Introduction

Wheat is one of the most widely cultivated crops in the world, covering an area of 219 million ha with a production of nearly 772 million t in 2017 (http://www.fao.org/faostat). Of the daily intake of humans, wheat provides 19% of the calories and 21% of the protein (http://www.fao.org/faostat). It is generally accepted that to match the global population demand wheat production will need to increase by 1.7% per year by 2050 [1]. Additionally, the increasing unpredictability of the weather conditions imposed by climate change will require the release of cultivars with very high yield potential that are able to maintain acceptable yield levels and stability under a broad range of environmental conditions. In the Mediterranean Basin, the climatic regions vary greatly, including both favourable lands and drylands that are subject to frequent drought episodes and high temperature stress, particularly during grain filling [2].

Wheat is estimated to have originated around 10000 years BP in the Fertile Crescent. From there it spread to the west of the Mediterranean Basin and reached the Iberian Peninsula around 7000 years BP [3]. During this migration, domestication and selection by humans resulted in the development of landraces that were very well adapted to local environments [4]. From the middle of the 20th century, the cultivation of local landraces was progressively abandoned, as a consequence of the Green Revolution, and they were replaced by the improved and more productive semi-dwarf cultivars. Mediterranean landraces are an important group of genetic resources because of their documented resilience to abiotic stresses, their resistance to pests and diseases, and their genetic diversity [4, 5].

Knowledge of genetic diversity provides valuable information for understanding the relationships between cultivars and facilitates their characterization and classification, determination of population structure, etc., thus enriching breeding strategies for crop improvement, for example helping breeders to develop new cultivars reducing pre-breeding activities. In the last few decades, several markers have been used for genetic studies [6]. However, the high-density genome coverage provided at low cost in recent years by new high-throughput genotyping technologies such as single nucleotide polymorphism (SNP) arrays or genotyping-by-sequencing (GBS) have made them the procedures of choice for wheat genetic analysis [7–11].

The aim of this study was to explore the existence of genetic and/or geographic structures and genetic diversity in collections of wheat landraces from the Mediterranean Basin and modern cultivars representative of the ones most widely cultivated in the region.

## Material and methods

### Plant material

With the aim of representing the past and current cultivated variability in the Mediterranean Basin, the plant material consisted of a germplasm collection of 354 bread wheat (*Triticum aestivum* L.) genotypes, the MED6WHEAT IRTA-panel, of which 170 correspond to landraces from 24 Mediterranean countries and 184 to modern varieties cultivated in 19 countries in the region (S1 File). The landraces were selected from a larger collection comprising 730 accessions of different origins on the basis of phenology, spike characteristics and passport data. Care was taken to include enough genotypes to represent the different climatic regions existing within the Mediterranean Basin [12]. Landrace populations were provided by public gene banks from Germany (IPK, Gatersleben), Italy (ISC, S. Angelo Lodigiano), Romania (Suceava GenBank, Suceava), Russia (VIR, St. Petersburg), Spain (CRF-INIA, Madrid), the Netherlands (CGN-WUR, Wageningen) and the USA (NSGC-USDA, Aberdeen, ID). Modern cultivars were provided by public institutions (CIMMYT, ICARDA, INRA and the University of Belgrade), breeding companies and the germplasm collection of the IRTA breeding programme. Accessions were bulk-purified during two cropping cycles to select the dominant type and seed was increased in plots in the same field to ensure a common origin for all lines.

### Molecular characterization

DNA isolation was performed from lyophilized leaf samples at Trait Genetics GmbH (Gatersleben, Germany). Accessions were genotyped with 13177 SNPs from the Illumina Infinium 15K Wheat SNP Chip at Trait Genetics GmbH (Gatersleben, Germany). Markers were ordered according to the SNP map developed by Wang et al. [13].

### Data analysis

Polymorphic information content (PIC) values were calculated following the formula described by Botstein et al. [14] using the Cervus software v3.0.7 [15] (available at http://www.fieldgenetics.com). Genetic diversity was estimated as total diversity (*H*_*T*_) [16] using POPGENE v1.32 [17]. The coefficient of genetic differentiation, i.e. the proportion of total variation that is distributed between populations (*G*_*ST*_), was calculated as *G*_*ST*_ *= D*_*ST*_*/H*_*T*_, where *D*_*ST*_ is the genetic diversity between populations. *D*_*ST*_ was calculated as *D*_*ST*_ *= H*_*T*_*—H*_*S*_, where H_S_ is the mean genetic diversity within populations. Gene flow was estimated as *Nm* = 0.5(1- *G*_*ST*_)/*G*_*ST*_ according to McDonald and McDermott [18].

Linkage disequilibrium (LD) was estimated as the square of marker correlations (*r*^*2*^) for markers with known map positions using TASSEL 5.0 [19] at a significance level of *P*<0.001 with a sliding window of 50 cM. The intra-chromosomal *r*^*2*^ values were plotted against the genetic distance and a LOESS curve was fitted to determine the distance at which the curve intercepts the line of a critical value of *r*^*2*^ in order to estimate how fast the LD decay occurs for each chromosome. The critical value of *r*^*2*^ was determined as the mean *r*^*2*^ for each genome.

The genetic structure of the collection was estimated using the Bayesian clustering algorithm implemented in the STRUCTURE software v2.3.4 [20] using an admixture model with burn-in and Monte Carlo Markov chain for 10000 and 100000 cycles, respectively. A continuous series of K were tested from 1 to 10 in seven independent runs. The Evanno method [21] was used to calculate the most likely number of subpopulations with STRUCTURE HARVESTER software [22]. Principal coordinates analysis (PCoA) based on genetic distance was performed using GenAlEx 6.5 [23].

Diversity analysis between accessions was determined by simple matching coefficient [24] implemented in DARwin software v.6 [25]. The un-rooted tree was calculated using the neighbour-joining clustering method [26]. The tree is divided in:

- Clusters: Main divisions of the tree
- Branches: Division within clusters
- Groups: Genotypes from the same subpopulation within a branch

## Results

### SNP polymorphism and diversity

A total of 11196 polymorphic markers were located in the map developed by Wang et al. [13]. In order to reduce the risk of errors in further analyses, markers and accessions were analysed for the presence of duplicated patterns and missing values. For the landrace collection, 8 markers with more than 25% of missing values as well as 730 markers with minor allele frequency (MAF) lower than 5% were excluded from the analysis, leaving a total of 10458 SNPs (Table 1). For the modern collection, 3 markers with more than 25% of missing values and 487 markers with an MAF lower than 5% were excluded, leaving 10706 polymorphic markers. The total number of polymorphic markers was 11074, of which 10090 (91%) were polymorphic in both collections, 368 only in landraces and 616 only in modern cultivars.

**Table 1:** Number of SNP markers (N), gene diversity (*H*_*T*_), polymorphic information content (PIC) and linkage disequilibrium (LD) (*r*^*2*^, % of markers in LD at *P*<0.001, and LD decay in cM) for all of the chromosomes and genomes in both types of germplasm.

	Landraces						Modern cultivars					
	N	*H*_*T*_	PIC	LD (*r*^*2*^)	LD (%)	LD decay	N	*H*_*T*_	PIC	LD (*r*^*2*^)	LD (%)	LD decay
1A	595	0.39	0.33	0.16	45	2	604	0.37	0.28	0.18	44	1
1B	812	0.36	0.30	0.11	35	2	834	0.33	0.28	0.17	45	2
1D	293	0.36	0.30	0.34	49	1	295	0.39	0.33	0.41	60	3
2A	576	0.38	0.30	0.13	30	1	590	0.36	0.30	0.16	42	3
2B	925	0.36	0.30	0.10	28	1	999	0.36	0.29	0.18	48	2
2D	376	0.37	0.30	0.30	59	2	388	0.40	0.33	0.40	68	2
3A	522	0.37	0.30	0.09	28	1	522	0.35	0.31	0.17	46	1
3B	770	0.35	0.30	0.10	31	1	781	0.34	0.30	0.14	45	2
3D	134	0.35	0.28	0.13	23	1	137	0.36	0.29	0.14	23	1
4A	435	0.35	0.30	0.10	26	1	433	0.35	0.29	0.16	43	1
4B	375	0.39	0.31	0.14	38	3	382	0.36	0.32	0.17	51	2
4D	39	0.25	0.21	0.09	23	2	51	0.39	0.30	0.12	32	2
5A	628	0.36	0.31	0.12	29	1	655	0.36	0.31	0.18	46	3
5B	860	0.38	0.31	0.11	31	2	867	0.37	0.31	0.18	53	2
5D	93	0.33	0.27	0.12	29	10	143	0.38	0.30	0.12	29	10
6A	648	0.36	0.30	0.14	34	7	674	0.36	0.31	0.18	52	9
6B	774	0.36	0.31	0.13	33	2	787	0.32	0.30	0.26	63	4
6D	135	0.35	0.28	0.18	45	1	146	0.40	0.32	0.15	38	4
7A	703	0.37	0.31	0.09	29	2	665	0.35	0.31	0.11	37	4
7B	652	0.37	0.30	0.11	34	2	637	0.36	0.31	0.17	56	2
7D	113	0.32	0.26	0.07	16	4	116	0.29	0.25	0.08	21	6
Genome A	4107	0.37	0.31	0.12	31	-	4143	0.36	0.30	0.16	43	-
Genome B	5168	0.37	0.31	0.11	31	-	5287	0.35	0.30	0.18	51	-
Genome D	1183	0.33	0.27	0.22	39	-	1276	0.37	0.30	0.20	44	-
Total	10458	0.36	0.30	0.14	32	-	10706	0.36	0.30	0.18	46	-

The D genome had the lowest number of markers, whereas the B genome showed the best coverage (Table 1). Genetic diversity (*H*_*T*_) and PIC were estimated for each chromosome. For the landraces, *H*_*T*_ ranged from 0.39 (1A, 4B) to 0.25 (4D) (mean 0.36) and PIC ranged from 0.33 (1A) to 0.21 (4D) (mean 0.30). For the modern collection, *H*_*T*_ ranged from 0.40 (2D, 6D) to 0.29 (7D) (mean 0.36) and PIC ranged from 0.33 (1D, 2D) to 0.25 (7D) (mean 0.30) (Table 1). A summary statistics is reported in S2 File.

### Linkage disequilibrium

LD was determined (*r*^*2*^) for single chromosomes. In the landraces, LD ranged from 0.07 in chromosome 7D to 0.34 in chromosome 1D, with a mean among chromosomes of 0.14. The percentage of locus pairs showing a significant LD at *P*<0.001 ranged from 16% to 59% for chromosomes 7D and 2D, respectively, with a mean of 33% (Table 1). In modern cultivars the mean value of *r*^*2*^ was 0.18, with 45% of the locus pairs in LD. Chromosome 7D, as reported for landraces, showed the lowest LD (0.08), with 21% of the locus pairs in LD at *P*<0.001 (Table 1). The maximum LD was found in chromosome 1D (0.41), showing 60% of the locus pairs with a significant LD at *P*<0.001. However, as reported for the landraces, the chromosome with the most locus pairs with a significant LD was 2D (68%, *r*^*2*^ = 0.4).

The extent of LD was also investigated for the three genomes. The highest *r*^*2*^ was found in the D genome for both landraces and modern cultivars (0.22 and 0.20, respectively). The D genome had the most markers with significant LD at *P*<0.001 (39%) in the landraces, whereas in the modern cultivars the B genome had the most, with 51% of the locus pairs showing a significant LD at *P*<0.001 (Table 1). The decay of LD varied for each chromosome. For the A and B genomes, LD decay ranged from 1 to 9 cM, whereas for the D genome it ranged from 1 to 10 cM (Table 1, S3 File).

### Population structure

The first analysis of the population structure of the 354 accessions was carried out using 557 common SNPs markers between landraces and modern cultivars evenly distributed across the genome. The markers were selected according to the distance of the LD decay for each chromosome in order to avoid the use of markers with a significant LD. The Bayesian clustering method using the Evanno test [21] to infer the most likely number of structured subpopulations (*ΔK*) revealed the presence of two distinct subpopulations, one including landraces and the other including modern cultivars (data not shown). On the basis of this result, a subsequent analysis of population structure was performed independently for the landraces and modern cultivars.

**Fig 1:**
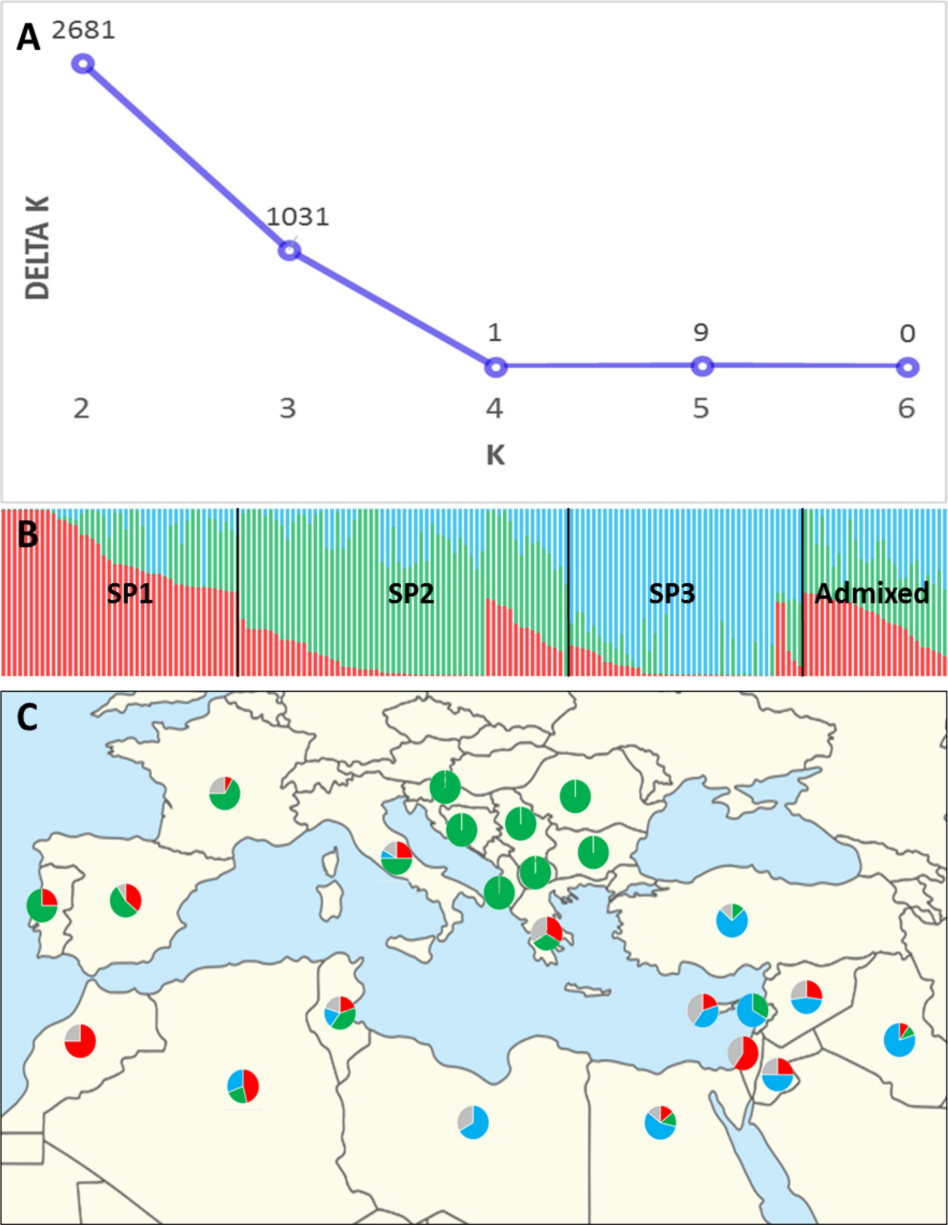
Genetic structure of Mediterranean landraces. (A) Estimation of the number of subpopulations (SPs) according to the Evanno test. (B) Inferred structure of the landrace collection based on 170 genotypes. Each individual is represented by a coloured bar with length proportional to the estimated membership to each of the three SPs. (C) Geographic distribution of the wheat subpopulations within the Mediterranean Basin. Circles indicate the proportion of each SP in the country. Red, SP1 (western Mediterranean); green, SP2 (northern Mediterranean); blue, SP3 (eastern Mediterranean); grey, admixture. Map source: https://commons.wikimedia.org/wiki/File:Middle_East_location_map.svg.

The highest value of *ΔK* for the landraces was observed for K = 2 (2861), followed by K = 3 (1031) (Fig 1A). Population structure for both K = 2 and K = 3 showed a geographical pattern. For K = 2 the landraces were separated following an east-west pattern within the Mediterranean Basin, whereas for K = 3 landraces mainly from the Balkan Peninsula were separated either from eastern Mediterranean ones or from French and Italian ones included in the western Mediterranean. The subpopulations (SPs) were classified as western (SP1), northern (SP2) and eastern Mediterranean (SP3) (Fig 1B) according to the geographical region of the countries most represented in the SP. The inferred population structure for K = 3 showed that 45% of the accessions (77 out of 170) showed a strong membership coefficient (*q-*value) to one of the SPs (*q*>0.7), whereas using a moderate *q*-value (>0.5) the number of accessions within an SP increased to 144 (85%), leaving 26 as admixed (S1 File). The western Mediterranean SP (SP1) included 43 accessions, of which 28 corresponded to western countries (Spain, Portugal, France, Morocco, Algeria and Tunisia), with 23 of them having a mean *q*-value greater than 0.8 (Table 2). SP1 also included 10 landraces (23%) from eastern countries and 6 (14%) from northern countries. The northern Mediterranean SP (SP2) included 59 accessions, with 68%, 24% and 8% corresponding to northern (Albania, Bosnia and Herzegovina, Croatia, France, Greece, Italy, Macedonia, Romania and Serbia), western and eastern Mediterranean countries, respectively (Table 2 and Fig 1B and 1C). Finally, the eastern Mediterranean SP (SP3) included 42 accessions, of which 86% corresponded to eastern countries (Cyprus, Egypt, Jordan, Iraq, Lebanon, Libya, Syria and Turkey) and 12% and 2% to western and northern countries, respectively (Table 2). As shown in Table 2 and Fig 1, the northern Mediterranean SP included accessions from Balkan countries, but also French, Italian and Spanish landraces. On the other hand, three accessions from Israel belonged to the western Mediterranean SP, but their *q*-values (average *q* = 0.605) were lower than those of the accessions from western Mediterranean countries. Also, 40% of the accessions from Tunisia belonged to SP2. Accessions from Portugal and Spain were distributed in SP1 and SP2. As shown in Table 2, the inconsistencies observed for these countries could be related to the low *q*-values for the most represented SP. The three Portuguese accessions included in SP2 had a mean *q*-value of 0.592, and the Spanish and Greek accessions showed *q*-values slightly higher than 0.5 for the two SPs (Table 2), denoting a high level of admixture in all of them.

**Table 2:** Mean membership coefficients across the landraces from each country included in each SP. Only structured genotypes (*q*>0.5) are shown in the table. *q-*values >0.7 are indicated in bold.

	SP1		SP2		SP3	
Country	*q* mean	N	*q* mean	N	*q* mean	N
Albania			**0.755**	3		
Algeria	**0.862**	6	**0.762**	3	0.601	4
Bosnia and Herzegovina			0.691	3		
Bulgaria			0.657	3		
Croatia			**0.700**	3		
Cyprus	0.661	1			0.501	2
Egypt	**0.992**	1	0.680	1	**0.706**	4
France	0.502	1	**0.707**	8		
Greece	0.565	2	0.540	2		
Iraq	0.517	1	0.546	1	**0.915**	8
Israel	0.605	3				
Italy	0.615	3	**0.727**	6	**0.986**	1
Jordan	0.614	1			**0.704**	2
Lebanon			0.676	1	**0.818**	2
Libya					**0.726**	2
Macedonia			**0.889**	4		
Morocco	**0.845**	15				
Portugal	**0.822**	1	0.592	3		
Romania			**0.774**	4		
Serbia			**0.761**	4		
Spain	0.526	4	0.544	6		
Syria	0.591	3			**0.966**	5
Tunisia	**0.973**	1	**0.738**	3	0.539	1
Turkey			0.552	1	**0.910**	11

SP1, western Mediterranean; SP2, northern Mediterranean; SP3, eastern Mediterranean.

**Fig 2:**
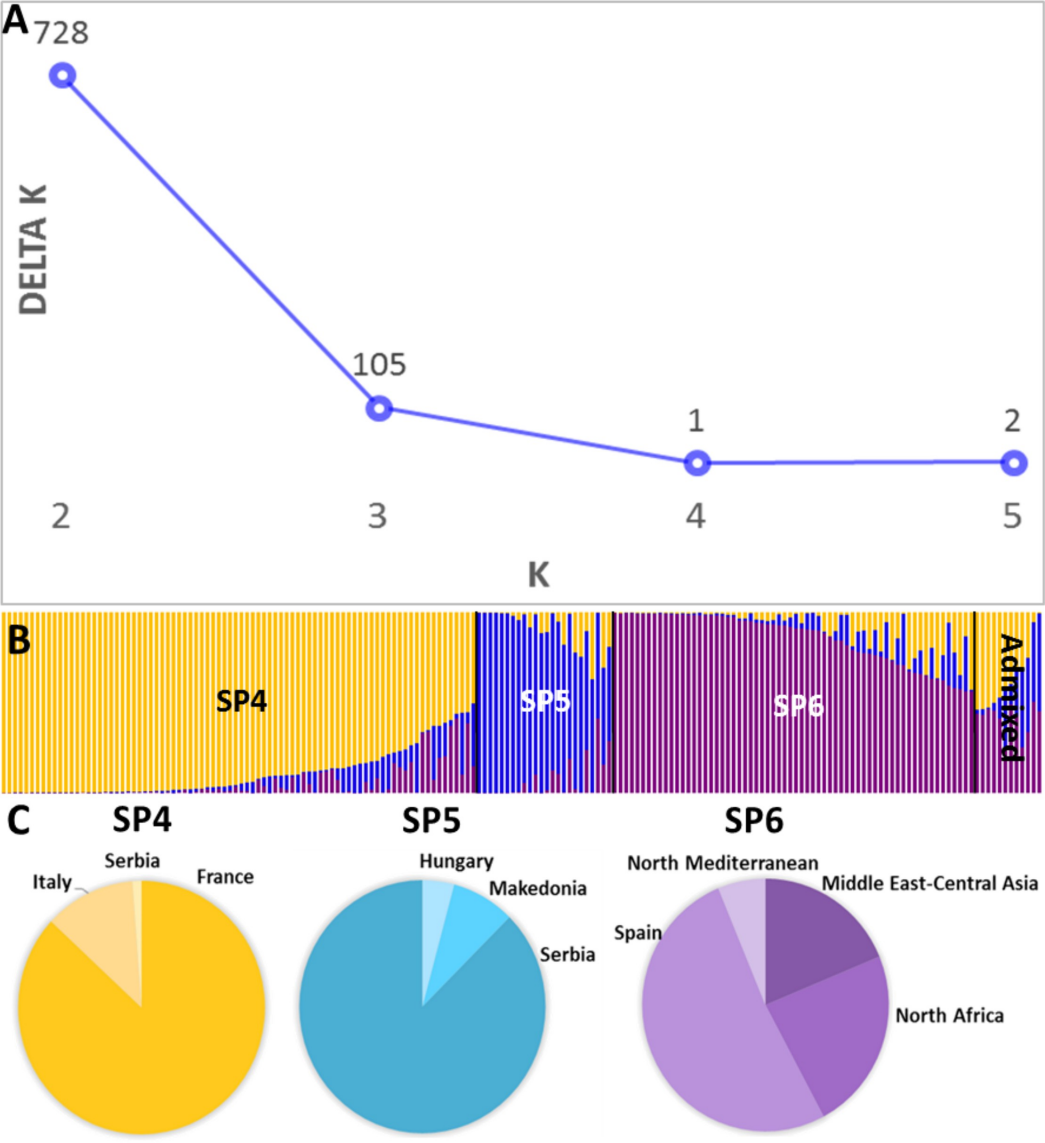
Genetic structure of the modern cultivars. (A) Estimation of the number of subpopulations (SPs) according to the Evanno test. (B) Inferred structure of the collection based on 184 genotypes. Each individual is represented by a coloured bar with length proportional to the estimated membership to each of the three subpopulations. (C) Proportion of cultivars from the different countries/regions within each SP. Yellow, SP4; blue, SP5; violet, SP6.

Following the Evanno test, as previously reported for landraces, the most likely number of structured SPs (*ΔK*) for modern cultivars was K = 2 (728) (Fig 2A). The first cluster grouped mainly cultivars developed by French and Italian breeding programmes, and the second one grouped mainly cultivars from North Africa, the Middle East and Spain, with evident CIMMYT and ICARDA genetic background (S1 File). A set of cultivars mainly from Serbia remained admixed. If the second highest value for the structured subpopulations was chosen (K = 3) (105), a third SP clustered all Serbian accessions, plus 2 from Macedonia and 1 from Hungary (Fig 2B and 2C, S1 File). According to the classification into three structured subpopulations, 79% of the accessions (145 out of 184) showed a *q* >0.7. The first SP (SP4) included 73 cultivars from France, 10 from Italy and 1 from Serbia. The second SP (SP5) included cultivars from eastern Europe, mainly Serbia (21 out of 24). The third SP (SP6) included 33 cultivars from Spain, 15 cultivars from North African countries, 12 from the Middle East and Central Asia (Afghanistan), and finally 4 from northern Mediterranean countries (France and Italy). Finally, 12 cultivars remained as admixed.

**Fig 3:**
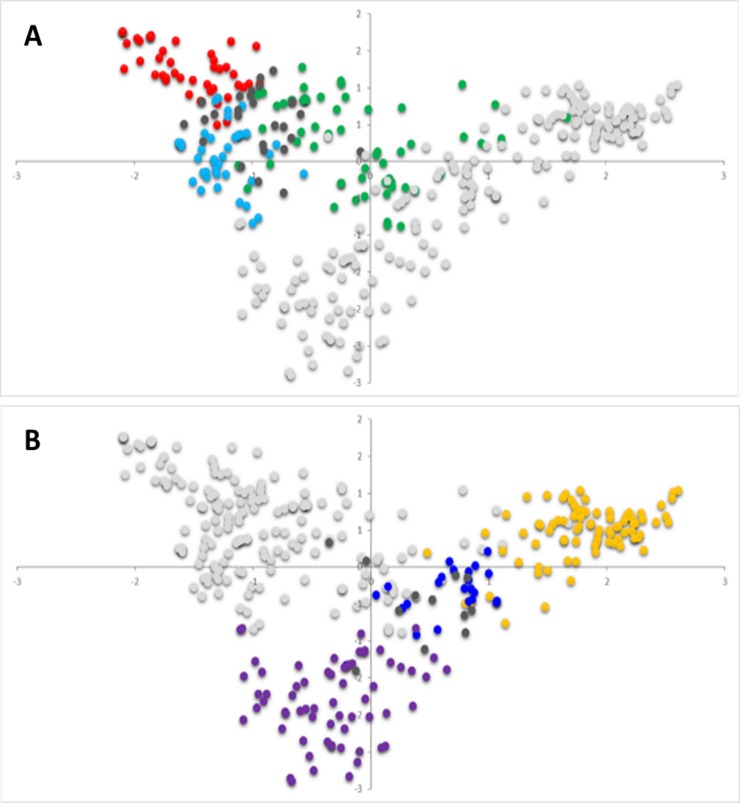
Principal coordinates analyses based on genetic distance. A) Landraces: red, SP1; green, SP2; blue, SP3; dark grey, admixed; light grey, modern cultivars. B) Modern cultivars: yellow, SP4; dark blue, SP5; violet, SP6; dark grey, admixed; light grey, landraces.

The relationships between landraces and modern cultivars were also analysed by PCoA as a complementary way to visualize their clusters. In agreement with the results shown by STRUCTURE, the first two coordinates of the PCoA clearly separated the landraces from the modern cultivars, and within each group accessions were clustered matching the results of STRUCTURE (Fig 3). Landraces from the Balkan Peninsula ‘TRI 1667’ and ‘TRI 1671’ (Albania), ‘Moriborska’ (Bosnia and Herzegovina), ‘41-II/4-B’ and ‘Legan Bezosja’ (Serbia), and ‘Solonetu Nou’ (Romania) were positioned on the positive side of the PCoA1 close to the origin of the axes, together with the modern Serbian cultivars. Additionally, two landraces from Italy (‘TRI 16900’ and ‘TRI 16516’) and three from France (‘Mounton a Epi Rouge’, ‘TRI 14046’ and ‘TRI 17938’) were located within the modern cultivars from SP4 that grouped modern French and Italian cultivars.

The six SPs showed a total genetic diversity (*H*_*T*_) ranging from 0.2651 for modern cultivars from the Balkans and eastern Europe (SP5) to 0.3690 for northern Mediterranean landraces (SP2) (Table 3). The genetic diversity among SPs (*D*_*ST*_) was low (0.0706), resulting in a genetic differentiation (*G*_*ST*_) among SPs of 0.1838. This means that only about 18% of the variability observed was due to differences between SPs. The estimation of *G*_*ST*_ also allowed us to estimate the gene flow (*Nm*) among SPs. The value of this estimate (2.22) indicates a high level of gene exchange, which denotes a low genetic differentiation among the SPs. When analysed by type of accessions, the landraces showed a *G*_*ST*_ = 0.1198 and an *Nm* = 3.67, whereas the modern cultivars showed a *G*_*ST*_ = 0.1673 and an *Nm* = 2.49, indicating higher gene exchange between the landrace SPs (Table 3). When comparisons were made between two SPs, gene flow ranged from 2.53 between western Mediterranean landraces (SP1) and modern Balkan cultivars (SP5) to 9.39 between northern Mediterranean landraces (SP2) and cultivars developed mainly by French and Italian breeding programmes (SP4) (Table 3).

**Table 3:** Genetic diversity and variation between the MED6WHEAT subpopulations (SPs).

	N	*H*_*T*_	*H*_*S*_	*D*_*ST*_	*G*_*ST*_	*Nm*
SP1	43	0.2873	-	-	-	-
SP2	59	0.3690	-	-	-	-
SP3	42	0.3132	-	-	-	-
SP4	82	0.2995	-	-	-	-
SP5	62	0.2651	-	-	-	-
SP6	24	0.3476	-	-	-	-
Total	312	0.3842	0.3136	0.0706	0.1838	2.22
Landraces	144	0.3672	0.3232	0.0440	0.1198	3.67
Modern	168	0.3652	0.3041	0.0611	0.1673	2.49
SP1-SP2	102	0.3594	0.3282	0.0312	0.0868	5.26
SP1-SP3	85	0.3363	0.3003	0.0360	0.1070	4.17
SP1-SP4	125	0.3452	0.2934	0.0518	0.1501	2.83
SP1-SP5	67	0.3307	0.2762	0.0545	0.1648	2.53
SP1-SP6	105	0.3690	0.3174	0.0516	0.1398	3.08
SP2-SP3	101	0.3737	0.3411	0.0326	0.0872	5.23
SP2-SP4	141	0.3521	0.3343	0.0178	0.0506	9.39
SP2-SP5	83	0.3628	0.3171	0.0457	0.1260	3.47
SP2-SP6	121	0.3844	0.3583	0.0261	0.0679	6.86
SP3-SP4	124	0.3549	0.3064	0.0485	0.1367	3.16
SP3-SP5	66	0.3445	0.2892	0.0553	0.1605	2.61
SP3-SP6	104	0.3304	0.2892	0.0412	0.1247	3.51
SP4-SP5	106	0.3176	0.2823	0.0353	0.1111	4.00
SP4-SP6	144	0.3658	0.3236	0.0422	0.1154	3.83
SP5-SP6	86	0.3594	0.3063	0.0531	0.1477	2.88

### Cluster analysis

**Fig 4:**
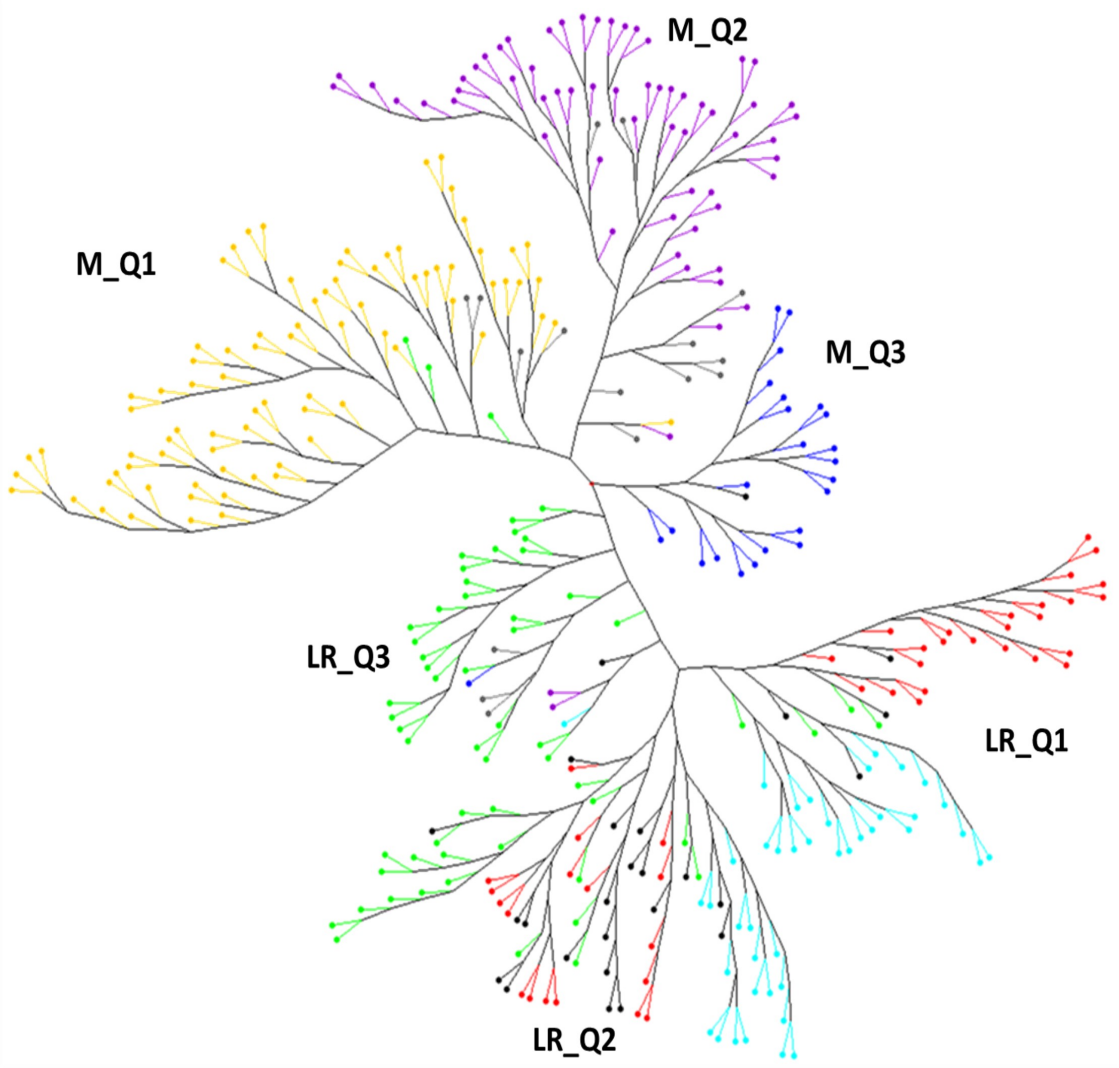
Un-rooted neighbour-joining dendrogram. Colours of branches correspond to the SPs obtained by STRUCTURE analysis. Landraces (LR): red, SP1; green, SP2; blue, SP3. Modern cultivars (M): yellow, SP4; dark blue, SP5; violet, SP6.

To better detail the kinship among accessions, a neighbour-joining tree was built using the common SNPs markers between the landraces and modern cultivars. The dendrogram showed two main clusters with a robust separation between them (Fig 4). Within each of these clusters, accessions were mainly grouped in agreement with the groups obtained previously by STRUCTURE and PCoA analysis (Fig 4).

The first cluster of modern cultivars (M_Q1) included cultivars from France and Italy (SP4), in addition to three landraces: ‘Bahatane’ and ‘TRI17938’ from France and ‘TRI16516’ from Italy. The second cluster of modern cultivars (M_Q2) represented mainly the accessions carrying CIMMYT and ICARDA genetic background (SP6), including cultivars developed in Turkey, Spain, Egypt, Syria, Morocco, Algeria, France, Italy, Afghanistan, Sudan and Tunisia. This cluster also included the French cultivar ‘Boticelli’ classified in SP4 but including 37% of the genetic background from SP6. Finally, the third cluster of modern cultivars (M_Q3) contained the group of all elite cultivars from the Balkan Peninsula and eastern Europe (SP5): Serbia (23), Macedonia (2) and Hungary (1). This cluster also included the Syrian landrace ‘Salamuni-A’, classified as admixed by STRUCTURE.

The clustering of landraces suggested a more complex distribution among the SPs according to the higher frequency of admixture revealed by STRUCTURE. The first cluster (LR_Q1) showed three branches, the first one with landraces mainly from the western Mediterranean (SP1), with Morocco as the best-represented country of the branch (45% of the accessions). The second country represented in this branch was Algeria (14%), and the remaining accessions from Cyprus, Egypt, Greece, Iraq, Portugal, Spain, Syria, Tunisia and Turkey were represented in a lower frequency (3%-6%). The second and third branches included mainly eastern Mediterranean landraces, with 81% of the accessions coming from Egypt, Iraq, Jordan, Syria and Turkey. The remaining accessions were from western (Algeria, 11% and Tunisia, 4%) and northern (France, 4%) Mediterranean countries.

The second cluster (LR_Q2) was represented by landraces from the three SPs in two main branches. The first branch included a division between genotypes from the eastern (SP3) and western (SP1) Mediterranean SPs. The eastern group was represented mainly by Turkish landraces (46%). An additional 27% was represented by landraces from Cyprus, Iraq, Jordan and Lebanon, and finally the remaining cultivars were previously grouped by structure analysis in the northern Mediterranean SP (SP2). The second group of the branch was composed mainly of landraces from the eastern Mediterranean countries Cyprus (10%), Israel (50%), Jordan (20%) and Syria (10%), although the Bayesian clustering determined that their structure was more similar to that of landraces from the western Mediterranean SP. Only one landrace from Morocco was included in this group. The second cluster included landraces from the western and northern Mediterranean SPs. The first branch included most of the accessions from western Mediterranean countries (Morocco, Portugal, Spain and Tunisia) and one accession from Libya. In this branch, 50% of the accessions showed high levels of admixture. The second branch of the cluster was divided into two groups, the first including mostly landraces classified as western Mediterranean by STRUCTURE. The group included cultivars from Algeria, Greece, Italy, Morocco and Turkey. The second group was mainly composed of northern Mediterranean landraces (71%) from Albania, France, Italy, Macedonia and Serbia. Although included by structure analysis in this SP, Portugal, Spain and Tunisia from the western Mediterranean and Lebanon from the eastern Mediterranean were also included in this group.

Finally, the remaining landraces were included in four branches (LR_Q3) of the main clusters examined above, with 76% corresponding to northern Mediterranean countries (Albania, Bulgaria, Bosnia and Herzegovina, France, Greece, Croatia, Italy, Romania and Serbia). Within these branches, five modern cultivars were also included: the Spanish cultivars ‘Montcada’ and ‘Montserrat’, the Turkish cultivars ‘Ata 81’ and ‘Cumhuriyet 75’, and the Serbian cultivar ‘KG 100’.

## Discussion

### Genetic diversity

Genetic diversity is essential for plant breeding because it provides new knowledge for improving cultivars. In wheat, the genetic diversity was narrowed down during the second half of the 20^th^ century as a consequence of the introduction of high-yielding improved semi-dwarf cultivars. Several studies, reviewed in Lopes et al. [27], have considered landraces to be a source of lost variability that can provide favourable genes to improve modern cultivars. However, in a recent study of the genetic structure of durum wheat Mediterranean landraces, Soriano et al. [6] reported that the great genetic distance estimated between modern cultivars and landrace populations denotes a low use of durum landraces by durum wheat breeding programmes. The knowledge of the genetic diversity within landrace populations will be of special interest for designing new crosses with commercial varieties in order to widening the variability of the new genotypes. As it is reported in Lopes et al. [27] the monitoring of the genetic diversity of landraces is an approach to increase the frequency of rare alleles in breeding programs to find new allelic variation of genes of interest.

Among the 11196 polymorphic markers in the MED6WHEAT panel with a known genetic position according to the map of Wang et al. [13], the B genome had the highest number of SNPs and the D genome the lowest, as reported by Alipour et al. [7] and Eltaher et al. [11]. The collection of Mediterranean landraces showed the lowest PIC and gene diversity values for the D genome, according to the findings of Lopes et al. [28]. Alipour et al. [7] and Eltaher et al. [11] related the low polymorphism in the D genome to the recent evolutionary history of this genome in comparison with the A and B genomes [29]. However, when the collection of modern cultivars was analysed, we found no differences between PIC values for the three genomes, while the D genome showed a slightly high gene diversity. Trethowan and Mujeeb-Kazi [30] and Jia et al. [31] concluded that higher diversity in the D genome may provide new elite and desirable alleles controlling important traits for dealing with climate change.

The average PIC value for both the landraces and the modern cultivars was 0.30. This value is in agreement with previous studies using bi-allelic markers such as SNP or DArT in either common or durum wheat. In common wheat, Lopes et al. [28] found a PIC value of 0.24 for the WAMI population genotyped with the 9K SNP array. Novoselović et al. [32] characterized a Croatian collection with 1229 DArT markers with an average PIC value among the populations of 0.30. Alipour et al. [7] genotyped a diversity panel of 369 Iranian landraces using 16506 GBS-based SNPs, reporting an average PIC of 0.172. El-Esawi et al. [33], using Austrian and Belgian wheats, found PIC values of 0.33 and 0.29, respectively, with 1052 DArT markers. Finally, Eltaher et al. [11], in an F3:6 Nebraska winter wheat population genotyped with 25566 SNPs generated by GBS, found a PIC value of 0.25. In durum wheat, similar PIC values have been reported (Baloch et al. [8], 0.26 and 0.30 using DArTseq and SNPs, respectively; Kabbaj et al. [10], 0.32). The SNP markers in our panel were moderately informative according to the classification of average PIC values into the three categories proposed by Botstein et al. [14]: highly informative (PIC>0.5), moderately informative (0.25<PIC<0.5) and slightly informative (PIC<0.25). Previous studies using SSR markers revealed higher levels of polymorphism. In a previous study of our group, Soriano et al. [6], using a panel of 192 durum wheat genotypes (mainly Mediterranean landraces) genotyped with 44 SSR markers, found an expected heterozygosity of 0.71. Similar results with SSRs have been reported in durum wheat [34, 35] and bread wheat [36–38]. The lower PIC value obtained with SNPs or DArTs than with SSR markers may be explained by their bi-allelic nature, which means that the maximum attainable PIC is 0.5 when the two alleles have the same frequency [11, 39].

### Linkage disequilibrium and population structure

Linkage disequilibrium is defined as the non-random association of alleles at different loci and decays rapidly with genetic distance. Thus, determining the LD decay over physical and genetic distance within a panel of genotypes is an important step for determining the resolution and marker density required for association studies. Moreover, LD is influenced by population structure due to stratification and unequal distribution of alleles within groups of genotypes, which can result in false associations [20].

In the current study, the mean *r*^*2*^ calculated for intra-chromosomal loci was 0.12 and 0.18 with 32% and 46% of the locus pairs in LD for landraces and modern cultivars, respectively. It is well known that crops gradually lose their genetic variability through domestication and breeding, resulting in more uniform cultivars, reducing the recombination rate and affecting LD [40, 41]. The D genome showed the highest *r*^*2*^ for both landraces and modern cultivars. Similar results were reported by Chao et al. [42] and Lopes et al. [28], who explained that higher LD in the D genome was linked to recent introgressions and population bottlenecks in the origin of hexaploid wheat.

The first attempt to dissect the genetic structure of the MED6WHEAT panel showed a clear separation based on historical breeding periods, i.e. landraces vs modern cultivars. Thus, for subsequent analysis of population structure, independent analyses were carried out for the two groups of germplasm. According to a Bayesian-based analysis, without a priori assignment of accessions to populations, the landraces showed a geographic structure according to the eastern and northern zones of the Mediterranean Basin, whereas accessions classified as western Mediterranean showed a high level of admixture. This classification denoted a migration from the centre of wheat domestication in the Fertile Crescent to the west of the Mediterranean Basin, as reported by Moragues et al. [43] and Soriano et al. [6] in durum wheat. The higher admixture found in western Mediterranean landraces may be due to the incorporation and fixation of favourable alleles from eastern and northern genetic pools during the migration process. By contrast, for modern cultivars the separation was mainly based on the pedigree of the accessions: CIMMYT/ICARDA, cultivars obtained mainly by French breeding programmes, and accessions from the Balkan Peninsula. These groups may have originated through the sharing of germplasm from different breeding programmes with similar growing conditions, particularly from the shuttle breeding carried out by international centres. For the landrace collection, only 45% of the accessions showed a strong *q*-value (>0.7), suggesting high levels of admixture among SPs, whereas for the modern cultivars 79% of the accessions showed a strong *q*-value. Oliveira et al. [44] suggested that admixture is the result of the incorporation of alleles from more than one gene pool because of the spread of wheat from different ancestral populations. Moragues et al. [43] proposed as a possible cause of admixture the exchange of germplasm between different Mediterranean regions during the expansion of the Arabian Empire. The low level of admixture between modern cultivars could be due to the development by breeding programmes of cultivars with specific adaptation to the local environments and the use of different genetic resources.

Based on the defined SPs, the results of PCoA and neighbour-joining were in agreement with those reported by STRUCTURE, showing first a robust separation between the landraces and modern cultivars, and within these main clusters a separation into three genetic SPs.

The origin of the axes in the PCoA showed a mixture between landraces and modern cultivars, as also reported by Oliveira et al. [45]. Landraces from the Balkan Peninsula co-localized with modern cultivars from the same region, and two landraces from Italy and three from France were located close to modern cultivars from those countries. A possible cause of this mixture could be the presence of these landraces in the pedigree of the modern cultivars, as reported for the French landrace ‘Mounton a Epi Rouge’, which according to Bonjean [46] played an important role in the pedigrees of improved French cultivars between 1965 and 1975.

According to the admixture revealed by STRUCTURE analysis, the modern cultivars showed well-defined clusters with differentiation among SPs in the neighbour-joining tree, whereas for the landraces the branches included accessions from different SPs. Only four genotypes from a given SP were misclassified within the clusters of modern cultivars. Within M_Q1, formed mainly by French and Italian cultivars, two French and one Italian landraces were also included. Cluster M_Q2, which grouped most cultivars carrying CIMMYT and ICARDA genetic background, also included the French cultivar ‘Boticelli’, which, although belonging to SP4, has an important genetic background (37%) from SP6. Cluster M_Q3 included a landrace from Syria, classified as admixed, with 30% of its genetic background from the northern Europe SP. The presence of landraces within modern cultivars was reported in a global durum wheat panel by Kabbaj et al. [10], who concluded that the simplest explanation was that they were not true landraces, but old tall cultivars wrongly labelled during the collecting mission by the gene banks. This seems a plausible explanation, considering that the first breeding attempts made by pioneer breeders or entrepreneurial Mediterranean agriculturalists consisted in identifying and isolating the best lines already existing within original wheat landraces [47]. Alternatively, the grouping within elite cultivars was probably due to the fact that they were used in breeding programmes to enlarge the genetic diversity, as reported for grain legumes by Sharma et al. [48].

When landraces were analysed by hierarchical clustering, a higher level of admixed genotypes was found on the basis of the STRUCTURE classification. Although the main groups within the clusters were formed mostly by members of specific SPs, a discrepancy between the classifying methods was observed among groups of the same cluster. As reported for clusters LR_Q1 and LR_Q2, some of the landraces misclassified by STRUCTURE according to their country of origin or with a high level of admixture were grouped by neighbour-joining into clusters containing accessions from the same geographical region. In LR_Q3, five modern cultivars were also grouped with landraces, probably due to the presence in their pedigree of genetic background from landraces or closely related accessions. However, the closed pedigree of most commercial cultivars did not allow us to clarify this.

These results highlight the importance of using different approaches to determine the genetic structure of a germplasm collection. Although the different methods are coincident for the genotypes with strong genetic membership to a given group, they are useful to complement the information provided when accessions show large admixture or gene flow among different geographical regions, as in the case of Mediterranean landraces.

### Gene flow

Genetic differentiation and gene flow provide information about population differentiation. Gene flow homogenizes populations by genetically decreasing variance among populations and increasing variance within populations. In our study, the analysis of genetic differentiation and gene flow indicated that the majority of the genetic variation was explained by differences among cultivars within genetic SPs. Differentiation of modern cultivar SPs was higher than that of landrace SPs, indicating a lower level of gene exchange among cultivars from different origins. These results are in agreement with population structure and neighbour-joining clustering, in which a higher level of admixture was found for landraces from different geographical regions. Accordingly, it has also been suggested that the low genetic differentiation among SPs is due to seed exchange by farmers, mainly influenced by geographic distances [49, 50]. When two SPs were compared, in general gene flow between landrace SPs was also higher than between modern cultivars. Within landraces, the highest gene flow was found between the northern Mediterranean SP and the western and eastern Mediterranean SPs. The value was lower for the exchange between the western and eastern Mediterranean SPs, supporting the hypothesis of geographic distance. When the modern cultivars were analysed, gene flow showed a higher value between SP4 (France and Italy accessions) and SP5 (Balkan accessions). Cultivars with a CIMMYT/ICARDA origin (SP6) had lower values of gene exchange with the other SPs, probably because of the delivery of improved inbred lines to be released by local programmes through the nurseries that these international centres distribute globally. In the case of modern cultivars, the SPs reflected similarities between the genetic pools managed by the breeding programmes conducted in each specific country. The highest gene flow value was reported between SPs from different periods, i.e. between landraces from the northern Mediterranean (SP2) and modern cultivars released by French and Italian breeding programmes (SP4), suggesting the presence of the genetic background of landraces or old cultivars in the improved modern varieties.

## Concluding remarks

The current study aimed to explore the presence of genetic and geographic structures in a collection of bread wheat landraces and modern cultivars representing the variability existing for the species in the Mediterranean Basin. The results demonstrated the usefulness of the methodologies employed for achieving this goal. The structure for landraces showed a geographical pattern with different levels of admixture, mainly justified by physical distances between the territories where they were collected, whereas the structure for modern cultivars reflected differences and similarities between the genetic pools managed by the breeding programmes operating in the region.

The results reported in the current study may be of special interest for driving the development of new cultivars with desirable traits for the climatic conditions of the Mediterranean Basin, and for identifying useful molecular markers through genome-wide association studies to assist breeding programmes.

## Supporting information

S1 File List of accessions. (DOCX)

S2 File Summary statistics for H_T_, PIC and LD for each one of the chromosomes. (XLSX)

S3 File Linkage disequilibrium plots. (TIF)
