# Supplementary material for: From landraces to improved cultivars: Assessment of genetic diversity and population structure of Mediterranean wheat using SNP markers
Source: PLoS One. 2019 Jul 15;14(7):e0219867. doi: 10.1371/journal.pone.0219867 (PMC6629082; doi:10.1371/journal.pone.0219867)
Supplement: S1 File — (DOCX) [file pone.0219867.s001.docx]

Supplementary material 1. List of accessions.

Landraces. SP: Subpopulation based on STRUCTURE

| **Accession** | **Country** | **SP** | **GenBank^1^** | **Accession No** |
| --- | --- | --- | --- | --- |
| TRI 1667 | Albania | SP2 | IPK | TRI 1667 |
| TRI 2100 | Albania | SP2 | IPK | TRI 2100 |
| TRI 1671 | Albania | SP2 | IPK | TRI 1671 |
| TRI 1313 | Bulgaria | SP2 | IPK | TRI 1313 |
| TRI 7819 | Bulgaria | SP2 | IPK | TRI 7819 |
| TRI 7821 | Bulgaria | SP2 | IPK | TRI 7821 |
| 408-IV/61 | Bosnia & Herzegovina | SP2 | NSGC | NSGC345409 |
| Moriborska | Bosnia & Herzegovina | SP2 | VIR | 22915 |
| Ranka | Bosnia & Herzegovina | SP2 | VIR | 38937 |
| TRI 10515 | Cyprus | Admixed | IPK | TRI 10515 |
| TRI 10526 | Cyprus | SP3 | IPK | TRI 10526 |
| TRI 10561 | Cyprus | Admixed | IPK | TRI 10561 |
| TRI 10590 | Cyprus | SP1 | IPK | TRI 10590 |
| TRI 10531 | Cyprus | SP3 | IPK | TRI 10531 |
| Ali Ben Makhloul | Algeria | SP3 | NSGC | NSGC48592 |
| Khalof | Algeria | SP3 | NSGC | NSGC67492 |
| Mahon Demias | Algeria | SP1 | NSGC | NSGC263419 |
| MG 17956 | Algeria | SP3 | NSGC | NSGC470815 |
| MG 17999 | Algeria | SP2 | NSGC | NSGC470851 |
| MG 18006 | Algeria | SP2 | NSGC | NSGC470857 |
| MG 18013 | Algeria | SP1 | NSGC | NSGC470864 |
| MG 18036 | Algeria | SP1 | NSGC | NSGC470884 |
| MG 18049 | Algeria | SP1 | NSGC | NSGC470895 |
| Bahatane | Algeria | SP3 | CGN | CGN06035 |
| Krelof - A | Algeria | SP2 | CGN | CGN11452 |
| Ble' du dahra-baal | Algeria | SP1 | VIR | 16152 |
| Mahon 7295 | Algeria | SP1 | ISC | T8801887 |
| Sachah | Egypt | SP2 | CGN | CGN06369 |
| Gibson | Egypt | SP3 | VIR | 21198 |
| Hauch | Egypt | SP3 | VIR | 20204 |
| Hindiffino crible | Egypt | Admixed | VIR | 21979 |
| Hindiffino non crible | Egypt | SP3 | VIR | 21982 |
| Mokhtar | Egypt | SP1 | VIR | 46124 |
| Hindi 62 | Egypt | SP3 | ISC | T8801285 |
| Bladette de Besplas | France | SP2 | NSGC | NSGC191706 |
| Ble Blanc de la Reole | France | SP2 | NSGC | NSGC48199 |
| Mars Rouge Sans Barbe | France | SP2 | NSGC | NSGC192398 |
| Touzelle Belle Abec | France | SP1 | NSGC | NSGC191709 |
| Touzelle Blanche Barbu | France | SP2 | NSGC | NSGC185381 |
| Touzelle Originario | France | Admixed | NSGC | NSGC191708 |
| Touzelle Rouge de Provence | France | Admixed | NSGC | NSGC184599 |
| Bladette de puylaurens | France | SP2 | CGN | CGN05384 |
| Mouton a epi rouge | France | SP2 | CGN | CGN05580 |
| Saisette | France | Admixed | CGN | CGN05661 |
| TRI 14046 | France | SP2 | IPK | TRI 14046 |
| TRI 17938 | France | SP2 | IPK | TRI 17938 |
| TRI 1425 | Greece | SP2 | IPK | TRI 1425 |
| TRI 1686 | Greece | SP1 | IPK | TRI 1686 |
| TRI 17989 | Greece | Admixed | IPK | TRI 17989 |
| TRI 2060 | Greece | SP1 | IPK | TRI 2060 |
| TRI 2071 | Greece | Admixed | IPK | TRI 2071 |
| TRI 2129 | Greece | SP2 | IPK | TRI 2129 |
| 986 | Croatia | SP2 | NSGC | NSGC264963 |
| Croatia 3 | Croatia | SP2 | NSGC | NSGC11225 |
| Croatia 6 | Croatia | SP2 | NSGC | NSGC11228 |
| Umarah | Iraq | SP1 | CGN | CGN06462 |
| TRI 15277 | Iraq | SP3 | IPK | TRI 15277 |
| TRI 15292 | Iraq | SP3 | IPK | TRI 15292 |
| TRI 16079 | Iraq | SP3 | IPK | TRI 16079 |
| TRI 16084 | Iraq | SP2 | IPK | TRI 16084 |
| TRI 11548 | Iraq | SP3 | IPK | TRI 11548 |
| TRI 16080 | Iraq | SP3 | IPK | TRI 16080 |
| TRI 11528 | Iraq | SP3 | IPK | TRI 11528 |
| TRI 16063 | Iraq | SP3 | IPK | TRI 16063 |
| TRI 8358 | Iraq | SP3 | IPK | TRI 8358 |
| CGN06182 | Israel | SP1 | CGN | CGN06182 |
| CGN06204 | Israel | SP1 | CGN | CGN06204 |
| CGN04191 | Israel | Admixed | CGN | CGN04191 |
| Palestinskaya | Israel | Admixed | VIR | 15818 |
| 17310 | Israel | SP1 | VIR | 17310 |
| Cappellina | Italy | SP2 | IPK | TRI 13007 |
| TRI 15321 | Italy | SP1 | IPK | TRI 15321 |
| TRI 14055 | Italy | SP2 | IPK | TRI 14055 |
| TRI 15226 | Italy | Admixed | IPK | TRI 15226 |
| TRI 16900 | Italy | SP2 | IPK | TRI 16900 |
| TRI 16895 | Italy | SP3 | IPK | TRI 16895 |
| TRI 14842 | Italy | SP1 | IPK | TRI 14842 |
| Solina | Italy | SP2 | IPK | TRI 13424 |
| TRI 15219 | Italy | Admixed | IPK | TRI 15219 |
| TRI 14173 | Italy | SP2 | IPK | TRI 14173 |
| TRI 16516 | Italy | SP2 | IPK | TRI 16516 |
| Cappelli | Italy | SP1 | IPK | TRI 16574 |
| 25 | Jordan | SP3 | NSGC | NSGC420932 |
| Dorziyeh Karak - B | Jordan | SP3 | NSGC | NSGC283147 |
| SY 271 | Jordan | Admixed | NSGC | NSGC487289 |
| 17411 | Jordan | SP1 | VIR | 17411 |
| Beyrouth 11 | Lebanon | SP3 | NSGC | NSGC278531 |
| Beyrouth 3 | Lebanon | SP2 | NSGC | NSGC278533 |
| Salamouni | Lebanon | SP3 | NSGC | NSGC182673 |
| TRI 17974 | Libya | Admixed | IPK | TRI 17974 |
| TRI 14643 | Libya | SP3 | IPK | TRI 14643 |
| TRI 14668 | Libya | SP3 | IPK | TRI 14668 |
| Canivano | Morocco | SP1 | CGN | CGN06294 |
| Fez 2 | Morocco | SP1 | CGN | CGN06049 |
| Recio | Morocco | SP1 | CGN | CGN06292 |
| CGN04157 - A | Morocco | Admixed | CGN | CGN04157 |
| CGN04158 | Morocco | SP1 | CGN | CGN04158 |
| CGN06246 | Morocco | SP1 | CGN | CGN06246 |
| CGN06247 | Morocco | SP1 | CGN | CGN06247 |
| CGN06248 | Morocco | SP1 | CGN | CGN06248 |
| CGN06252 | Morocco | SP1 | CGN | CGN06252 |
| CGN06255 | Morocco | SP1 | CGN | CGN06255 |
| CGN06260 | Morocco | Admixed | CGN | CGN06260 |
| CGN06264 | Morocco | SP1 | CGN | CGN06264 |
| CGN06266 | Morocco | Admixed | CGN | CGN06266 |
| CGN06269 | Morocco | SP1 | CGN | CGN06269 |
| CGN06271 | Morocco | Admixed | CGN | CGN06271 |
| CGN06284 | Morocco | Admixed | CGN | CGN06284 |
| CGN06297 | Morocco | SP1 | CGN | CGN06297 |
| TRI 18287 | Morocco | SP1 | IPK | TRI 18287 |
| TRI 18291 | Morocco | SP1 | IPK | TRI 18291 |
| TRI 18308 | Morocco | SP1 | IPK | TRI 18308 |
| 309-VII/33 | Makedonia | SP2 | NSGC | NSGC345309 |
| 340-VII/45 | Makedonia | SP2 | NSGC | NSGC345340 |
| VII/1-B | Makedonia | SP2 | NSGC | NSGC362589 |
| Stara bela | Makedonia | SP2 | CGN | CGN04172 |
| Magueija | Portugal | SP2 | CRF | BGE012669 |
| Santareno | Portugal | SP2 | CRF | BGE011900 |
| Temporao de coruche | Portugal | SP2 | CRF | BGE012846 |
| Tremes branco | Portugal | SP1 | CRF | BGE012703 |
| Bistra | Romania | SP2 | Suceava | SVGB5538 |
| Pades | Romania | SP2 | Suceava | SVGB7916 |
| Solonetu nou | Romania | SP2 | Suceava | SVGB14976 |
| SVGB10195 | Romania | SP2 | Suceava | SVGB10195 |
| Raton de Belalcazar | Spain | SP1 | CRF | BGE011825 |
| Cabezorro | Spain | SP1 | CRF | BGE011882 |
| Negrete de Cañaveras | Spain | SP2 | CRF | BGE012132 |
| Pelon blanco | Spain | Admixed | CRF | BGE012196 |
| Chamorro de Villadiego | Spain | SP2 | CRF | BGE012205 |
| Blat petit de Olot | Spain | SP1 | CRF | BGE012870 |
| Candeal | Spain | SP2 | CRF | BGE012392 |
| Isla de Fuerteventura | Spain | SP2 | CRF | BGE013760 |
| Hembrilla de Jerga | Spain | SP2 | CRF | BGE018232 |
| Extremo Sur Argelino | Spain | SP2 | CGN | CGN05749 |
| Xeixa Tarragona | Spain | SP1 | CRF | BGE018242 |
| 41-II/4-B | Serbia | SP2 | NSGC | NSGC345043 |
| Crvenica | Serbia | SP2 | NSGC | NSGC184168 |
| Piskulja | Serbia | SP2 | NSGC | NSGC184188 |
| Legan bez osja | Serbia | SP2 | VIR | 38803 |
| 401 | Syria | SP1 | NSGC | NSGC94569 |
| Aleppo 21 | Syria | Admixed | NSGC | NSGC278540 |
| Aleppo 28 | Syria | SP3 | NSGC | NSGC278545 |
| Aleppo 32 | Syria | SP3 | NSGC | NSGC278547 |
| Aleppo 33 | Syria | SP3 | NSGC | NSGC278548 |
| Damaskus 12 | Syria | SP3 | NSGC | NSGC278537 |
| Damaskus 8 | Syria | SP1 | NSGC | NSGC278536 |
| K1140 | Syria | Admixed | NSGC | NSGC253959 |
| Kaundouhari | Syria | SP3 | NSGC | NSGC182711 |
| TRI 8375 | Syria | SP1 | IPK | TRI 8375 |
| Salamuni - A | Syria | Admixed | VIR | 17172 |
| Allorca | Tunisia | SP2 | CGN | CGN05358 |
| Sbei noir | Tunisia | SP1 | CGN | CGN06378 |
| TRI 17006 | Tunisia | SP3 | IPK | TRI 17006 |
| TRI 17002 | Tunisia | Admixed | IPK | TRI 17002 |
| Florence 193 | Tunisia | SP2 | ISC | T8800968 |
| 763 | Turkey | Admixed | NSGC | NSGC119302 |
| 1170 | Turkey | Admixed | NSGC | NSGC119309 |
| 811 (B) | Turkey | SP3 | NSGC | NSGC119305 |
| 1552 | Turkey | SP2 | NSGC | NSGC119325 |
| 2103 | Turkey | SP3 | NSGC | NSGC119348 |
| 2933 | Turkey | SP3 | NSGC | NSGC119366 |
| 2936 | Turkey | SP3 | NSGC | NSGC119369 |
| Edirne | Turkey | SP3 | NSGC | NSGC111244 |
| Gemir - B | Turkey | SP3 | NSGC | NSGC166257 |
| Kirmizi kiluk - A | Turkey | SP3 | NSGC | NSGC165149 |
| Ormece | Turkey | SP3 | NSGC | NSGC166545 |
| Saribasak | Turkey | SP3 | NSGC | NSGC165146 |
| T-317 | Turkey | SP2 | NSGC | NSGC109368 |
| Yazlik | Turkey | SP3 | NSGC | NSGC165115 |
| Yumusak | Turkey | SP3 | NSGC | NSGC165160 |

^1^CGN: Centre for Genetic Resources, Wageningen, The Netherlands; CRF, Centro Nacional de Recursos Fitogenéticos, Madrid, Spain; IPK: Gene Bank for Agricultural and Horticultural Crop Species, Gatersleben, Germany; ISC: Istituto Sperimentale per la Ceralicoltura, Sant’angelo Lodigiano, Italy; NSGC: National Small Grains Collection, Aberdeen, ID, USA; Suceava: Suceava GenBank, Romania; VIR: Vavilov Institute of Plant Genetic Resources, St. Petersburg, Russia.

Modern cultivars. SP: Subpopulation based on STRUCTURE

| **cultivar** | **Country** | SP | **Pedigree^1^** |
| --- | --- | --- | --- |
| Takhar 96 | Afghanistan | SP6 | Veery-7/Opata-m-85 |
| Ain abid | Algeria | Admixed |  |
| Adelaide | Canada | Admixed |  |
| Misir-2 | Egypt | SP6 | Super-Kauz/Baviacora-92 |
| Misir-1 | Egypt | SP6 | Oasis-86/Super-Kauz//4*Bacanora-88/3/2*Pastor |
| Gemmeiza-10 | Egypt | SP6 | Maya-74/Olesen//1160-47/3/Bluebird/g11/4/Chat/5/Crow |
| Sakha-69 | Egypt | SP6 | Inia-f-66/rl-4220//Siete-cerros-t-66/Yaqui-50 |
| Sids-12 | Egypt | SP6 | Buckbuck//Siete-cerros-66/Alondra/5/Maya-74/Olesen//1160.147/3/Bluebird/Gallo/4/Chat/6/Maya-74/Vulture//Cmh-74-a-63014/Super-x |
| Gemmeiza-11 | Egypt | SP6 | Bobwhite/Kvs//Siete-cerros-66/Seri-82/3/Giza-168/Sakha-61 |
| Sahel-1 | Egypt | SP6 | ns-732/Pima/Veery |
| Sids 1 | Egypt | SP6 | hd-2172/Pavon//1158-57/Maya-74 |
| Adagio | France | SP4 |  |
| Candelo | France | SP4 |  |
| Aviso | France | SP4 | Moisson/Topaze |
| Belsito | France | SP4 |  |
| Innov | France | SP4 | Ordeal/Sidereal |
| Andalou | France | SP4 |  |
| Fiorenzo | France | SP4 | Rabd-88-13/Virlor |
| Sensas | France | SP4 | S-0179/S-32203 |
| Adhoc | France | SP4 |  |
| Charles peguy | France | SP4 | Thatcher/Vilmorin-27//Ariana |
| Trocadero | France | SP4 | Baroudeur/Bercy |
| Astral | France | SP4 | Fortunato/Yga/3/Florence aurore//g-4 |
| Aerobic | France | SP4 |  |
| Bramante | France | SP4 | Victo/Soissons |
| Soissons | France | SP4 | Iena(Jena)/(hybride-naturel)hn-35 |
| Isengrain | France | SP4 | Apollo,deu/Soissons |
| Soberbio | France | SP4 |  |
| Cipres | France | SP4 |  |
| Bologna | France | SP4 | H-89092/H-89136//Soissons |
| Avelino | France | SP4 |  |
| Viriato | France | SP4 |  |
| Nogal | France | Admixed | Norrona/Gasser |
| Premio | France | SP4 |  |
| Diamento | France | SP4 |  |
| Rgt Somontano | France | SP4 |  |
| Lazaro | France | SP4 |  |
| Altamira | France | SP4 | 96248/Isengrain |
| Andino | France | SP4 |  |
| Arezzo | France | SP4 |  |
| Solehio | France | SP4 | Isengrain/Ornicar |
| Mecano | France | SP4 |  |
| Guadalete | France | SP6 |  |
| Bonpain | France | SP6 | Prinqual/Cornette |
| Equilibre | France | SP4 |  |
| Soledad | France | SP4 |  |
| Tremie | France | SP4 | s-32/Moulin |
| Bastide | France | SP4 | Fertil/Arche |
| Aubusson | France | SP4 | Tremie/91-b-294 |
| Garcia | France | SP4 |  |
| Kumberri | France | SP4 |  |
| Akim | France | SP4 |  |
| Camargo | France | SP4 |  |
| Exotic | France | SP4 | Etecho/Vivant |
| CCB Ingenio | France | SP4 |  |
| Bueno | France | SP4 |  |
| Sublim | France | SP4 |  |
| Alhambra | France | SP4 |  |
| Bandera | France | SP4 |  |
| Inoui | France | SP4 | Charly/Victo |
| SY Moisson | France | SP4 | Cappelle-desprez//hybride-80-3/Etoile-de-choisy |
| Raffy | France | SP4 |  |
| Aguila | France | SP4 |  |
| Rodrigo | France | SP4 | Aztec/Legion |
| Sollario | France | SP4 |  |
| Alpino | France | SP4 |  |
| Galpino | France | SP4 |  |
| Carles | France | SP4 |  |
| Sorrial | France | SP4 |  |
| Royssac | France | SP4 |  |
| Rimbaud | France | SP4 |  |
| Rvalo | France | SP4 |  |
| Sobbel | France | SP4 |  |
| Sofru | France | SP4 |  |
| Apache | France | SP4 | Axial/nrpb-84-4233 |
| Illico | France | SP4 | Ormil/Apache |
| Galopin | France | SP4 |  |
| Sobred | France | SP4 |  |
| Sokal | France | SP4 |  |
| Cezanne | France | SP4 | Thesee/87-b-29 |
| Craklin | France | SP4 | 87-b-15/d-136 |
| Paledor | France | SP4 |  |
| Botticelli | France | SP4 | Perico/95-b-343 |
| Eureka | France | SP4 | Mironovskaya-808/Maris-huntsman/3/Vpm-1/Moisson(r-1-5-2)//Courtot |
| MV Emese | Hungary | SP5 | mv-ma/mv-12//f-2098-w-2-21 |
| Masaccio | Italy | SP4 | Oratorio/Genio |
| Zanzibar | Italy | SP4 | Frelon/61601//Capnor/Parador |
| Palesio | Italy | SP4 | Pandas/Recital |
| Toskani | Italy | SP4 |  |
| Trofeo | Italy | Admixed | Bolero/Mieti |
| Anapo | Italy | SP6 | eg-52/bel-118 |
| Andana | Italy | SP4 | Unknown/Eridano |
| Anforeta | Italy | SP6 | eg-83/bel-118 |
| Arabia | Italy | SP4 | Guadalupe/Tibet |
| Carisma | Italy | Admixed | d-29/f-65 |
| Agape | Italy | SP4 | Serio/Tremie |
| Antille | Italy | SP4 |  |
| Tiepolo | Italy | SP4 | Oracle/Calodine |
| Abate | Italy | SP4 | eg-52/Eridano |
| Arz | Lebanon | SP6 | Mayo-54-e/Lerma-rojo-64//Tacuari/3/Lerma-rojo-64//Tezanos-pintos-precoz/Yaqui-54 |
| Olga | Makedonia | SP5 |  |
| Balkania | Makedonia | SP5 |  |
| Siete cerros | Mexico | SP6 |  |
| Marchouch 8 | Morocco | SP6 |  |
| Aguilal | Morocco | SP6 | Sais*2/ks-85241-14 |
| Achtar | Morocco | SP6 | Hork/Yamhill//Kalyansona/Bluebird |
| Nesma | Morocco | SP6 |  |
| Arrehane | Morocco | SP6 |  |
| KG100 | Serbia | SP5 |  |
| PKB Arena | Serbia | SP5 |  |
| Ana Morava | Serbia | SP5 | Morava/Una |
| PKB Lepoklasa | Serbia | SP5 |  |
| PKB Ratarica | Serbia | SP5 |  |
| Zvezdana | Serbia | SP5 | ns-63-27//Stamena/ns-Rana-5 |
| PKB Vizeljka | Serbia | SP5 |  |
| Simonida | Serbia | SP5 | ns-63-25//Rodna/ns-3288 |
| BG Merkur | Serbia | SP5 |  |
| BG Carica | Serbia | SP5 |  |
| PLB Talas | Serbia | SP5 |  |
| BG Vitka | Serbia | SP5 |  |
| Vizija | Serbia | SP5 | Kozara/Skopjanka |
| PKB Mlinarka | Serbia | SP5 |  |
| Zlatna | Serbia | SP5 | Jasenica/Rodna |
| Aleksandra | Serbia | SP5 |  |
| Planeta | Serbia | SP5 | Fillo-9/Yazi-6//Rabudo-1/Shag-14 |
| Pobeda | Serbia | SP5 | Sremica/Balkan |
| Aurelia | Serbia | SP5 |  |
| Zemunska rosa | Serbia | SP5 | Skopljanka/Proteinka |
| Renesansa | Serbia | SP5 | Yugoslavia/ns-55-25 |
| Kruna | Serbia | Admixed |  |
| NS 40S | Serbia | SP4 |  |
| Chambo | Spain | SP4 |  |
| 08THES2162 | Spain | SP6 |  |
| Vejer | Spain | SP6 |  |
| Antequera | Spain | SP6 |  |
| Conil | Spain | SP6 | Croc-1/(205)tr.ta//Borlaug-m-95/3/2*Milan |
| Marchena | Spain | SP6 | Croc-1/(205)tr.ta//Borlaug-m-95/3/2*Milan |
| Tejada | Spain | SP6 | Chilero/Parula//Baviacora-92/3/Milan/Kauz |
| Babui | Spain | SP6 |  |
| Catedral | Spain | SP6 |  |
| Eneas | Spain | SP6 |  |
| Califa sur | Spain | SP6 |  |
| Cartaya | Spain | SP6 | Kavkaz/Buho//Kalyansona/Bluebird |
| Escacena | Spain | SP6 | Seri-82/Rayon-89 |
| Jerezano | Spain | SP6 | Thornbird//Maya-74/Nacozari-76/3/Rabe/4/Milan |
| Galeon | Spain | SP6 |  |
| Kilopondio | Spain | SP6 |  |
| Trebujena | Spain | SP6 | Shearwater/Yavaros |
| Victorino | Spain | SP6 |  |
| Alcala | Spain | SP6 |  |
| Rinconada | Spain | SP6 |  |
| Yecora | Spain | SP6 | Ciano-67//Sonora-64/Klein-Rendidor/3/ii-8156 |
| Cielo | Spain | SP6 |  |
| Gazul | Spain | SP6 |  |
| Galera | Spain | SP6 |  |
| Mapeña | Spain | SP6 | Tr-353/Betres//Alcotan/3/Rinconada/4/3*Betres |
| Marca | Spain | SP6 |  |
| Anza | Spain | SP6 | Lerma-rojo-64//Norin-10/Brevor/3/3*Andes-enano |
| Odiel | Spain | SP6 | Br-5237/Cavalier |
| Trimax | Spain | SP6 |  |
| Algido | Spain | Admixed |  |
| Artur Nick | Spain | SP6 |  |
| Mulhacen | Spain | SP6 |  |
| Platero | Spain | SP6 |  |
| Adalid | Spain | Admixed |  |
| Dollar | Spain | SP6 |  |
| Montcada | Spain | Admixed |  |
| Montserrat | Spain | Admixed | Damiano/Montjuich |
| Idalgo | Spain | SP4 |  |
| Santoyo | Spain/France | SP4 |  |
| Debeira | Sudan | SP6 | hd-2160/5/Tobari-66/Ciano-67//Bluebird/3/Nainari-60*2//Tom-Thumb/Sonora-64/4/hd-1954 |
| Valbona | Switzerland | Admixed |  |
| Cham-8 | Syria | SP6 | Jupateco-f-73/Bluejay//Ures-81 |
| Cham-6 | Syria | SP6 | w-3918-a/Jupateco-73 |
| Babaga-3 | Syria | SP6 |  |
| Cham-4 | Syria | SP6 | Flicker/Hork |
| Hamam-4 | Syria | SP6 |  |
| Attila | Tunisia | SP6 |  |
| Karatopak | Turkey | SP6 | Tesia-79/Veery//Seri-82 |
| Ata 81 | Turkey | SP6 | Kavkaz/Ciguena |
| Cumhuriyet 75 | Turkey | SP6 | Sonora-64*2//Tezanos-pintos-precoz/Yaqui-54/3/Andes-64-a/4/2*Frocor//Yaqui/Kentana |
| Efe | Turkey | SP6 |  |
| Mane Nick | Turkey | SP6 |  |
| Gönen | Turkey | Admixed | 8156-Reselection/Mara//Bluebird |

^1^Pedigree from <http://genbank.vurv.cz/wheat/pedigree/pedigree.asp> and <http://wheatpedigree.net/>
