# Supplementary figures and images for: From landraces to improved cultivars: Assessment of genetic diversity and population structure of Mediterranean wheat using SNP markers
Source: PLoS One. 2019 Jul 15;14(7):e0219867. doi: 10.1371/journal.pone.0219867 (PMC6629082; doi:10.1371/journal.pone.0219867)

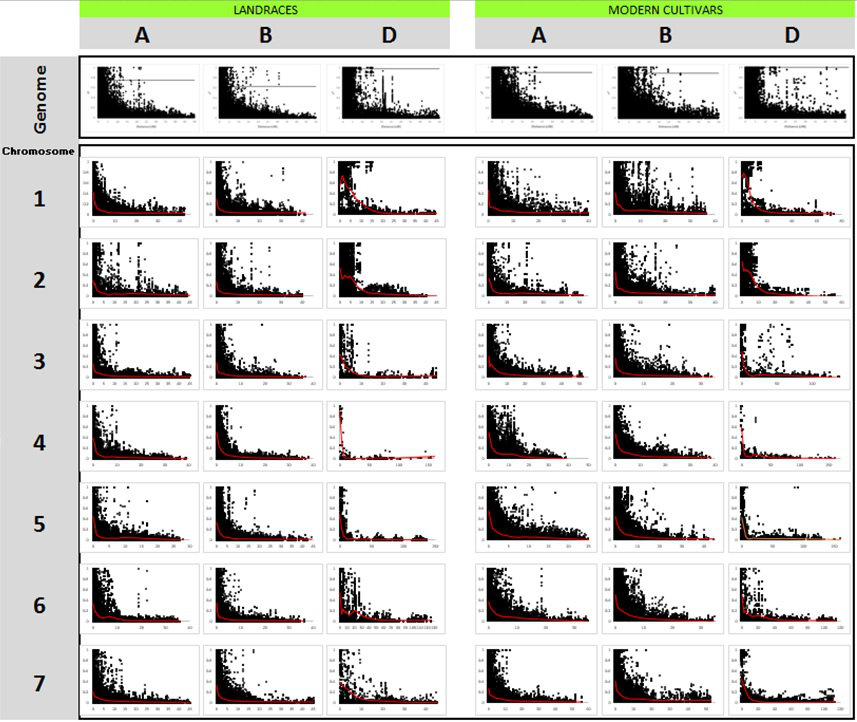

Supplement: S3 File — (TIF) [file pone.0219867.s003.tif]
